# Monitoring the SARS-CoV-2 Pandemic: Prevalence of Antibodies in a Large, Repetitive Cross-Sectional Study of Blood Donors in Germany—Results from the SeBluCo Study 2020–2022

**DOI:** 10.3390/pathogens12040551

**Published:** 2023-04-02

**Authors:** Ruth Offergeld, Karina Preußel, Thomas Zeiler, Konstanze Aurich, Barbara I. Baumann-Baretti, Sandra Ciesek, Victor M. Corman, Viktoria Dienst, Christian Drosten, Siegfried Görg, Andreas Greinacher, Marica Grossegesse, Sebastian Haller, Hans-Gert Heuft, Natalie Hofmann, Peter A. Horn, Claudia Houareau, Ilay Gülec, Carlos Luis Jiménez Klingberg, David Juhl, Monika Lindemann, Silke Martin, Hannelore K. Neuhauser, Andreas Nitsche, Julia Ohme, Sven Peine, Ulrich J. Sachs, Lars Schaade, Richard Schäfer, Heinrich Scheiblauer, Martin Schlaud, Michael Schmidt, Markus Umhau, Tanja Vollmer, Franz F. Wagner, Lothar H. Wieler, Hendrik Wilking, Malte Ziemann, Marlow Zimmermann, Matthias an der Heiden

**Affiliations:** 1 Robert Koch Institute, Nordufer 20, 13353 Berlin, Germany; 2 German Red Cross Blood Service West, 58097 Hagen, Germany; 3 Institute for Immunology and Transfusion Medicine, University Medicine Greifswald, Sauerbruchstrasse, 17475 Greifswald, Germany; 4 Haema AG, Landsteinerstraße 1, 04103 Leipzig, Germany; 5 Institute for Medical Virology, German Centre for Infection Research, External Partner Site Frankfurt, University Hospital, Goethe University Frankfurt am Main, 39120 Frankfurt am Main, Germany; 6 Institute of Virology, German National Reference Laboratory for Coronavirus, Charité—University Medicine Berlin, 10117 Berlin, Germany; 7 Institute of Transfusion Medicine, University Hospital of Schleswig-Holstein, Lübeck/Kiel, Ratzeburger Allee 160, 23538 Lübeck, Germany; 8 Institute of Transfusion Medicine and Immunohaematology/Blood Bank, University Hospital Magdeburg, Leipziger Str. 44, 39120 Magdeburg, Germany; 9 Institute for Transfusion Medicine, University Hospital Essen, Hufelandstraße 55, 45147 Essen, Germany; 10 Institute of Transfusion Medicine and Immunohematology, German Red Cross Blood Transfusion Service Baden-Württemberg—Hessen, Sandhofstraße 1, 60528 Frankfurt am Main, Germany; 11 Bavarian Red Cross Blood Service, Herzog-Heinrich-Str. 2, 80336 München, Germany; 12 German Red Cross Blood Service NSTOB, Eldagsener Straße 38, 31832 Springe, Germany; 13 Institute of Transfusion Medicine, University Medical Center Hamburg-Eppendorf, 20251 Hamburg, Germany; 14 Center for Transfusion Medicine and Haemotherapy, University Hospital Giessen and Marburg, Langhansstr. 7, 35392 Giessen, Germany; 15 Institute for Transfusion Medicine and Gene Therapy, Faculty of Medicine, Medical Center—University of Freiburg, Hugstetter Str. 55, 79106 Freiburg, Germany; 16 IVD Testing Laboratory, Paul Ehrlich Institute, 63225 Langen, Germany; 17 Heart and Diabetes Centre NRW, Institute for Laboratory and Transfusion Medicine, Ruhr-University Bochum, 32545 Bad Oeynhausen, Germany

**Keywords:** SARS-CoV-2, COVID-19, seroprevalence, COVID-19 serological testing, blood donors, surveillance

## Abstract

SARS-CoV-2 serosurveillance is important to adapt infection control measures and estimate the degree of underreporting. Blood donor samples can be used as a proxy for the healthy adult population. In a repeated cross-sectional study from April 2020 to April 2021, September 2021, and April/May 2022, 13 blood establishments collected 134,510 anonymised specimens from blood donors in 28 study regions across Germany. These were tested for antibodies against the SARS-CoV-2 spike protein and nucleocapsid, including neutralising capacity. Seroprevalence was adjusted for test performance and sampling and weighted for demographic differences between the sample and the general population. Seroprevalence estimates were compared to notified COVID-19 cases. The overall adjusted SARS-CoV-2 seroprevalence remained below 2% until December 2020 and increased to 18.1% in April 2021, 89.4% in September 2021, and to 100% in April/May 2022. Neutralising capacity was found in 74% of all positive specimens until April 2021 and in 98% in April/May 2022. Our serosurveillance allowed for repeated estimations of underreporting from the early stage of the pandemic onwards. Underreporting ranged between factors 5.1 and 1.1 in the first two waves of the pandemic and remained well below 2 afterwards, indicating an adequate test strategy and notification system in Germany.

## 1. Introduction

In January 2020, the first severe acute respiratory syndrome coronavirus type 2 (SARS-CoV-2) infections were identified in Germany [1]. To date, more than 38.3 million cases and as of 30 March, 170,727 deaths have been reported via the statutory notification system in Germany [2]. Most individuals with a SARS-CoV-2 infection develop measurable antibodies, as well as those who are vaccinated. Seroprevalence studies can therefore help identify those infections missed by mandatory reporting and support decisions on infection control measures. The majority of seroprevalence studies performed addressed certain regions or a specific part of the population and were limited to a single sampling or a few time points [3,4,5,6,7]. Repetitive and representative sampling of the general population was very difficult in times of social distancing and lockdown. Therefore, early on in the pandemic, we decided to analyse residual blood donation specimens, as they were readily available in a substantial quantity and over a long period of time and could be tested efficiently. Blood donor-based serosurveillance has been a part of the strategy to monitor the pandemic in various countries [8,9,10,11,12,13]. More than 300 seroprevalence studies among blood donors have been identified in 39 countries or territories, with a total of more than seven million analysed specimens [14]. Seroprevalence estimates ranged from 0.1% in New Zealand in December 2020 to 100% in Scotland in May 2022 [15]. Our SeBluCo (Serological Investigation of Blood (German: Blut) Donors for SARS-CoV-2-Antibodies) study was conducted as a repetitive cross-sectional study for one year from late April 2020 to April 2021, supplemented by additional single cross-sectional samples in September 2021 and April/May 2022. Using that approach, we were able to monitor the proportion of SARS-CoV-2 antibodies in blood donors aged 18 years and older. In order to interpret the findings as a proxy for the presence of antibodies in the healthy general adult population aged 18–65 years, we adjusted for differences in demographic data between donors and the general population. The estimated seroprevalence can also be used to assess the degree of underreporting in other surveillance systems especially due to undiagnosed oligo- or asymptomatic infections.

## 2. Materials and Methods

In this study, we included residual specimens of blood donations from the participating blood establishments (BE) in Germany from April 2020 to April 2021, September 2021, and April/May 2022. The final date of collection was 18 May 2022. Participation began in some BE as early as 27 April 2020, and by 8 June 2020, specimens from all participating BE could be included.

Five infection waves were identified in Germany during the study period [16]. These were determined using a variety of parameters as suggested by the Pandemic Influenza Severity Assessment Tool (PISA) of the World Health Organisation (WHO) [17]. They included for example data on reported infections, data from the syndromic surveillance, mitigation measures, data on treatments, use of tests and relevant holidays as well as genomic data. The characteristics of the infection waves are shown in Table 1.

**Figure 1:**
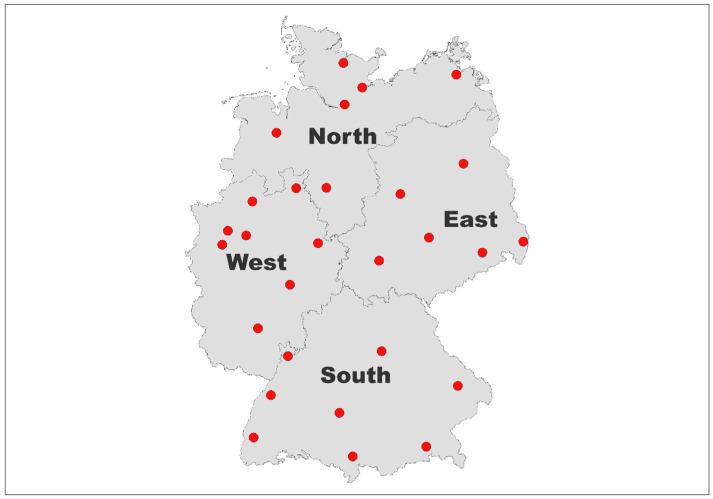
Geographic distribution of BE participating in the SeBluCo study.

Specimens were collected using convenience sampling techniques. All donors were symptom-free at donation. Donor selection guidelines mandated that after a resolved coronavirus disease (COVID)-19 infection, donors had to be deferred for four weeks. SARS-CoV-2 vaccination did not result in deferral. Donors who were explicitly invited to donate SARS-CoV-2 convalescent plasma were excluded. The BE collected specimens in 28 catchment areas (CA) in 14 of the 16 federal states. For the analysis, data were evaluated within the four marked larger regions North, East, South, and West (Figure 1).

In each of the 28 CA of the BE in 2020 and until April 2021, roughly 170 specimens were collected every two weeks, leading to approximately 10,000 specimens every month. Another cross-sectional sample with roughly 170 specimens/CA was collected in calendar weeks (CW) 36 and 37 in September 2021. In another sample in 2022, roughly 500 specimens were collected in each CA. Due to the effects of the pandemic on personnel and supply, regular sampling and/or testing was not feasible for all BE at all time points. In addition, the study was initially planned to last until October 2020 and the prolongation also caused uncertainties which led to a lower number of specimens in late 2020.

The contributing BE were:

- German Red Cross Blood Service West (4 CA)
- University Medicine Greifswald (1 CA)
- University Hospital Magdeburg (1 CA)
- University Hospital Giessen and Marburg (1 CA)
- University Hospital Hamburg Eppendorf (1 CA)
- University Hospital Essen (1 CA)
- Medical Center University of Freiburg (1 CA)
- University Hospital of Schleswig-Holstein (2 CA)
- Bavarian Red Cross Blood Service (4 CA)
- German Red Cross Blood Transfusion Service Baden-Württemberg—Hessen, Frankfurt (6 CA)
- Haema AG (3 CA)
- German Red Cross Blood Service NSTOB (2 CA)
- Institute for Laboratory and Transfusion Medicine, Heart and Diabetes Centre NRW (1 CA).

### 2.1. Laboratory Testing

All specimens were tested for antibodies against the S1 domain of the spike protein (S1 antibodies) using the semiquantitative Anti-SARS-CoV-2-ELISA (IgG) (Euroimmun, Lübeck, Germany) according to the manufacturer‘s instructions. Specimens with an extinction ratio of ≥1.1 were considered positive. Initial ELISA testing was conducted either at the Robert Koch Institute (RKI) (6 BE, representing 14 CA) or at the respective BE. All testing material was provided by the RKI. The sensitivity and specificity of the test were determined for the specific batch used in the study by the Testing Laboratory for in vitro Diagnostics at the Paul Ehrlich Institute to be 83.03% and 99.65%, respectively [18]. 

From January 2021 onwards, S1-positive specimens were additionally screened for antibodies against the nucleocapsid (N) antigen (N antibodies) using the Roche Elecsys^®^ N-total antibody assay (Roche Diagnostics, Mannheim, Germany) in order to discriminate antibodies acquired by infection from vaccine-induced antibodies. In Germany, only vaccines that used spike protein antigens had a market authorisation. This testing was performed by the central laboratory of the German Red Cross Blood Service West according to the manufacturer’s instructions. The sensitivity and specificity of the test were derived from an analysis of the Testing Laboratory for in vitro Diagnostics at the Paul Ehrlich Institute (95.07% sensitivity and 100% specificity) [18]. Specimens with a cutoff index (COI) of ≥1.0 were viewed as positive. S1-positive specimens with N antibodies were considered a consequence of infection with SARS-CoV-2 (with or without additional vaccination), whereas antibodies against the S1 protein only were defined as vaccine-induced. In 2022, the naturally occurring waning of N antibodies after infection and the widespread vaccination made a laboratory-based decision on the nature of detected antibodies more difficult, and the proportion of N antibodies can only represent the minimum infection-induced prevalence.

Most (96.8%, *n* = 4634) of the positive specimens received until April 2021 were frozen at −30 °C and additionally analysed in a plaque reduction neutralisation assay (PRNT) (*n* = 647) and/or a surrogate virus neutralisation test (sVNT) (*n* = 4246) targeting the Angiotensin Converting Enzyme (ACE) 2 receptor binding domain of the SARS-CoV-2 spike protein (cPass™ Neutralization Antibody Detection Kit, Genscript Biotech, Piscataway Township, NJ, USA). A subset of 379 N-positive specimens collected in 2022 was analysed with discriminatory sVNT targeting the wild-type virus or the Omicron variant (cPass™ Neutralization Antibody Detection Kit, Genscript Biotech, Piscataway Township, NJ, USA). The sVNT was performed at the RKI according to the manufacturer’s instructions [19,20] without a serial dilution. Inhibition values of ≥30% were regarded as positive [21].

PRNT was carried out as described elsewhere [20]. PRNT was performed at the National Consultant Laboratory for Coronaviruses, the Institute of Virology at the Goethe University Frankfurt or at the RKI following the same protocol using VERO E6 cells (#85020206, European Collection of Authenticated Cell Cultures (ECACC), Porton Down, UK). Samples were analysed qualitatively using dilution titres of 20 and 80. Specimens in which a dilution titre of at least 20 resulted in a plaque reduction of 50% were considered positive.

### 2.2. Demographic Data, Case Numbers and Vaccination Coverage Rates

BE provided demographic data for each specimen, including year of birth, sex, and 3-digit postcode. Sex was only recorded as male and female as blood donor selection in Germany requires a binary classification of sex. The specimens and the data were fully anonymised at the site of collection. SARS-CoV-2 infections identified by PCR were reportable and obtained from the COVID-19 dashboard provided by the RKI [2]. Data on vaccinations were taken from the national COVID-19 Electronic Vaccination Coverage Monitoring database (Digitales Impfquotenmonitoring, DIM) of the German Federal Ministry of Health [22]. Vaccinations had to be reported on a daily basis, including a variety of parameters such as age group (5–11 years, 12–17 years, 18–59 years and 60+ years), sex, type of vaccine, vaccination date, and number of previous vaccinations. To account for delayed antibody response, dates of infection or vaccination were shifted backwards by 3 weeks in order to allow for comparisons of identified antibodies and reported cases or vaccinations.

### 2.3. Statistical Methods

We estimated the seroprevalence in periods of four weeks in different regions of Germany stratified by sex and age group. We included the age groups 18–29, 30–49, and 50–65 years. Due to the relatively low number of specimens from individuals aged 66 or older, seroprevalence estimates using the method described below were restricted to these age groups. Data on crude seroprevalence for all age groups can be found in the Supplementary Material (Table S2).

To compare the vaccine-induced seroprevalence with the vaccination rates according to the anonymised immunisation registry DIM, we also analysed the group of 18–59-year-old donors.

We defined the four greater geographical regions South (Bavaria and Baden-Württemberg, 27.4% of specimens), West (Northrhine-Westfalia, Rhineland Palatinate, Saarland, Hesse, 29.3% of specimens), East (Berlin, Brandenburg, Thuringia, Saxony, Saxony-Anhalt, 19.3% of specimens), and North (Bremen, Lower Saxony, Hamburg, Schleswig-Holstein, Mecklenburg Western Pomerania, 24.0% of specimens) of Germany, each including six to eight CA.

Each CA of the BE was assigned a level two Nomenclature des Unités Territoriales Statistiques (NUTS2) where the majority of contributing donors resided. Blood donors of the respective CA from other NUTS2 areas were interpreted as commuters and allocated to the assigned NUTS2, reflecting the seroprevalence of their assumed work or study area. The exemption from this strategy was the CA Breitscheid (German Red Cross Blood Service West), which contributed two NUTS2, as well as the whole of Bavaria (Bavarian Red Cross Blood Service), which was divided into North and South, each contributing to two NUTS2 areas. Details of the methodology can be found in the Supplementary Material.

The outcome of the study was the infection- and vaccination-induced SARS-CoV-2 seroprevalence over time and stratified for sex, age group, and region:

- Total SARS-CoV-2 seroprevalence was defined as the prevalence of specimens with S1 antibodies.
- In 2020, the prevalence of S1 antibodies indicated infection-induced SARS-CoV-2 seroprevalence in the absence of vaccinations. In 2021 and 2022, after the beginning of vaccinations, infection-induced seroprevalence was defined as the prevalence of specimens with both S1 and N antibodies.
- Vaccination-induced seroprevalence was defined as total seroprevalence minus infection-induced seroprevalence after the introduction of vaccinations.

Seroprevalence estimates were further adjusted for imperfect test accuracy according to a Bayesian framework with an a priori beta distribution based on observed sensitivities and specificities over time [18,23]. 

The underreporting factor was defined as the ratio between the estimated infection-induced seroprevalence, and the cumulative number of reported cases divided by the population of the respective combined CA.

Statistical analyses were performed using Stata version 17 (Stata Statistical Software: Release 17. College Station, TX, USA: StataCorp LLC) and R version 4.1.2. The Bayesian model for the adjustments was created using the R package “prevalence” [24].

## 3. Results

Overall, we tested 134,510 blood donation specimens: 74,978 specimens came from male donors (55.7%) and 59,527 from female donors (44.3%). For 5 specimens, information about sex was missing. Donor age ranged from 18–83 years, with a median age of 38 years. For 14 specimens, information about age was missing. Information on 3-digit postcode was missing for 39 specimens.

The monthly number of specimens contributed by all BE ranged from 937 in CW 15–18, 2020 to 14,034 in CW 17–20, 2022. The number of collected specimens over time is provided in Table S1 in the Supplementary Material.

### 3.1. Total Study-Wide Seroprevalence over Time

**Figure 2:**
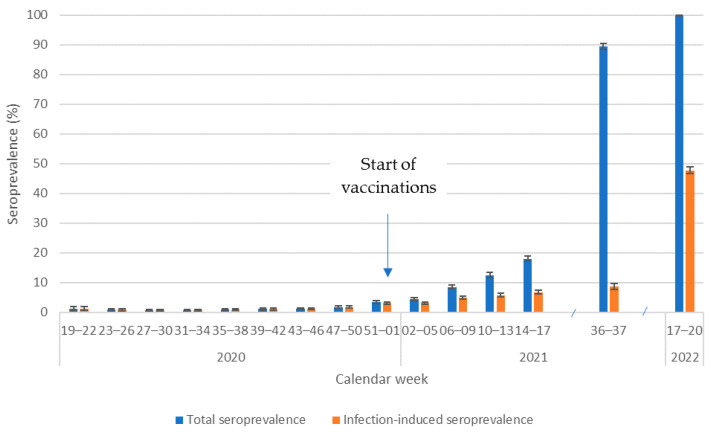
Adjusted total infection-induced seroprevalence over time.

In CW 19–22 (May) 2020, the total adjusted seroprevalence was 1.2% (95% CI: 0.6–2.1%). It then fell to 0.6% (95% CI: 0.4–0.9%) in CW 27–34 (July–August). Afterwards, it rose again but stayed below 2% until CW 47–50 (December), where it reached 1.8% (95% CI: 1.4–2.2%). At the end of 2020, the total seroprevalence started to rise and reached 18.1% (95% CI: 17.2–19.0%) in CW 14–17 (April) 2021. In CW 36–37 (September) 2021, total seroprevalence was estimated to be 89.4% (95% CI: 88.4.3–90.4%) and in CW 17–20 (April/May) 2022 100% (95% CI: 98.5–100) (Figure 2).

On 27 December 2020, COVID-19 vaccination began in Germany, prioritising the most vulnerable individuals, health professionals, and caretakers. According to the study definition, specimens with antibodies against S1 and N were considered to be infection-induced. From January 2021 to April 2021, the infection-induced seroprevalence estimate increased from 3.2% (95% CI: 2.7–3.6) to 6.8% (95% CI: 6.2–7.4). The infection-induced seroprevalence reached 8.6% (95% CI: 7.7–9.6) in September 2021 and 47.7% (95% CI: 46.6–48.8) in April/May 2022 (Figure 2).

Neutralising antibodies were detectable in 74% (3,429) of the 4634 tested ELISA-positive specimens in 2020 and 2021, varying from 57.1% in October 2020 to 80.3% in February 2021. In 2022, a subset of 379 specimens with N antibodies was analysed with a discriminatory surrogate neutralisation assay: 97.1% (368) of the specimens had neutralising antibodies against the wild-type virus and 92.9% (352) against the omicron variant. 

### 3.2. Seroprevalence in Different Regions

**Figure 3:**
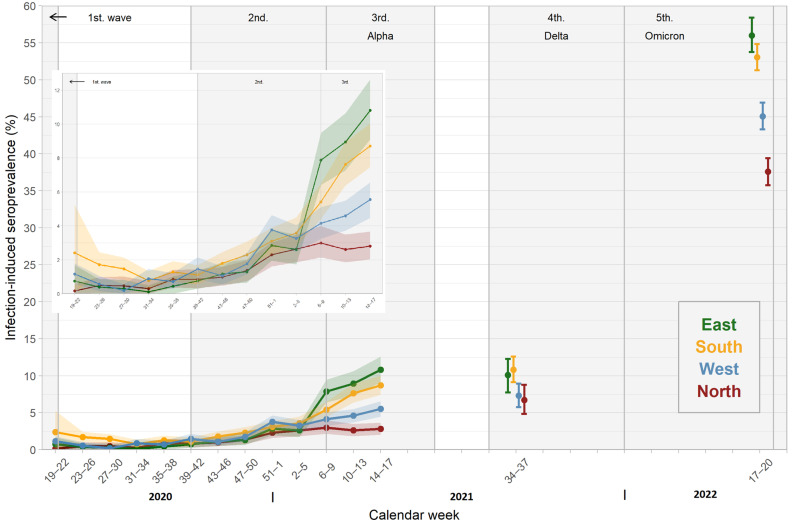
Infection-induced seroprevalence in different regions May 2020–April 2021, including a more detailed view on data from April 2020–April 2021.

The infection-induced seroprevalence varied over time in the different SeBluCo study regions (Figure 3). In the North, infection-induced seroprevalence remained low over time, ranging from 0.2–3.0% in the first year of the study. In the same time period, the greatest increase in seroprevalence was observed in the East, ranging from 0.1–10.8%. Seroprevalence was higher in the South after the first wave of the pandemic and reached 8.7% in April 2021. In the West, the increase in prevalence was not very pronounced, reaching 5.6% in April 2021. In September 2021, infection-induced seroprevalence was highest in the South (10.8%, 95% CI: 9.1–12.6) and the East (10.1%, 95% CI: 7.7–12.3) and lower in the West (7.3%, 95% CI: 5.8–8.9) and the North (6.7%, 95% CI: 4.8–8.8). In April/May 2022, infection-induced seroprevalence was highest in the East (56.0%, 95% CI: 53.8–58.4) and the South (53.1%, 95% CI: 51.3–54.9), lower in the West (45.1%, 95% CI: 43.3–46.9), and lowest in the North (37.6%, 95% CI: 35.8–39.4).

After the introduction of vaccinations, total seroprevalence also varied slightly in the study regions from January to April 2021. In September 2021, however, total seroprevalence was significantly different between the regions, with the lowest seroprevalence in the East (87.4%, 95% CI: 75.5–81.4) and higher in the South (89.6%, 95% CI: 85.9–91.3), the North (90.9%, 95% CI: 88.8–92.7), and highest in the West (93.6%, 95% CI: 92.1–95.1).

In April/May 2022, total seroprevalence amounted to 99.1–100% in all four regions.

### 3.3. Seroprevalence Stratified by Sex and Age 

#### 3.3.1. Sex

Total seroprevalence estimates for men and women did not differ significantly until CW 06/2021. In the CW 06–17/2021, the total seroprevalence estimates for women were up to a factor of 1.5 higher than estimates for men. In September 2021 and April/May 2022, seroprevalence was almost identical in male and female donors (88.8% and 88.2% and 99.9% and 100%, respectively). Data are shown in Table 2, including the proportion of male and female vaccinated study participants.

#### 3.3.2. Age

**Figure 4:**
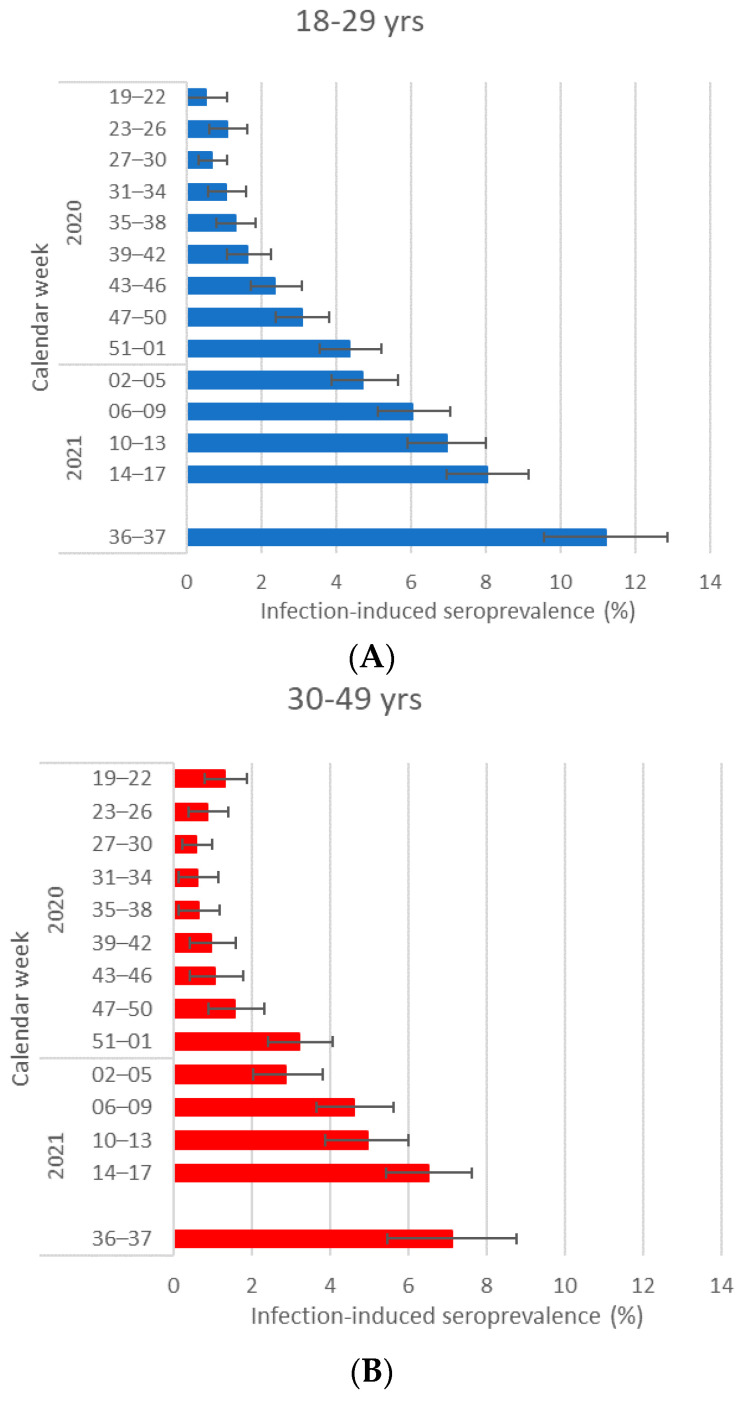

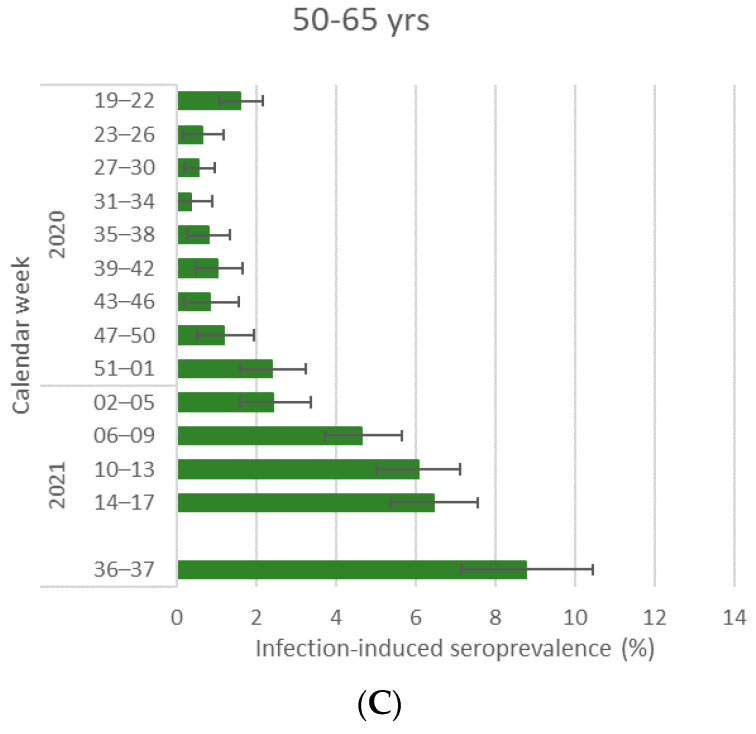
(**A**–**C**) Infection-induced seroprevalence in different age groups 18–29 (**A**), 30–49 (**B**), and 50–65 (**C**) years in 2020 and 2021.

Except for the first month of the study, the highest infection-induced seroprevalence estimates were found in donors aged 18–29 years. Data for 2020 and 2021 are shown in Figure 4A–C. Vaccine-induced antibodies did not vary significantly between the age groups. In 2022, infection-induced seroprevalence was 56.7% (95%CI: 54.9–58.5) in 18–29-year-old, 48.8% (95% CI: 47.1–50.4) in 30–49-year-old, and 41.8 (95% CI: 39.6–42.9) in 50–69-year-old donors. Information on crude prevalence for all specimens, including those aged >65 years, is available in Table S2 in the Supplementary Material.

### 3.4. Comparison of Reported Vaccinations with Vaccine-Induced Seroprevalence

**Figure 5:**
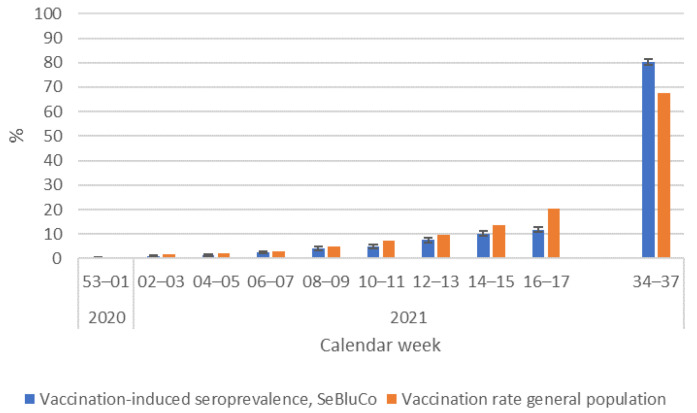
Vaccine-induced seroprevalence of the SeBluCo study participants and vaccination rate in the general population according to the anonymised immunisation registry DIM, 18–59-year-old individuals, 2020–2021.

The comparison of reported vaccinations in the general population and the prevalence of S1-only antibodies in the study population showed no significant differences from late December 2020 to April 2021. In September 2021, significantly more donors aged 18–59 years had S1 antibodies only, which we attribute to vaccination (80.3%, 95% CI: 79.1–81.4) compared to the vaccinations in the general population (65.4%) that were recorded in the anonymised immunisation registry DIM (Figure 5).

### 3.5. Comparison of Cumulative Case Reporting Rate and Infection-Induced Seroprevalence

**Figure 6:**
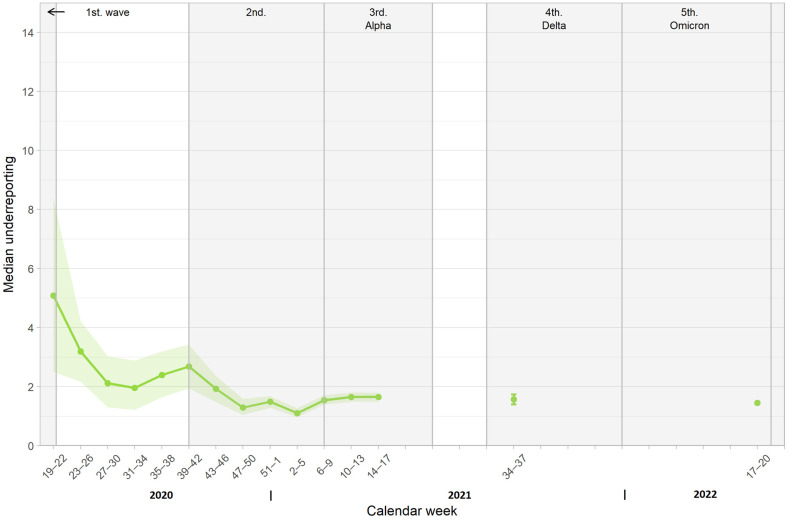
Estimated number of SARS-CoV-2 infections per reported COVID-19 case (underreporting) over time.

Based on infection-induced seroprevalence estimates, the estimated number of SARS-CoV-2 infections per reported COVID-19 case decreased from 5.1 (95% CI: 2.5–8.4) at the beginning of the study to 1.4 (95% CI: 1.4–1.5). Figure 6 shows the degree of underreporting over time.

**Figure 7:**
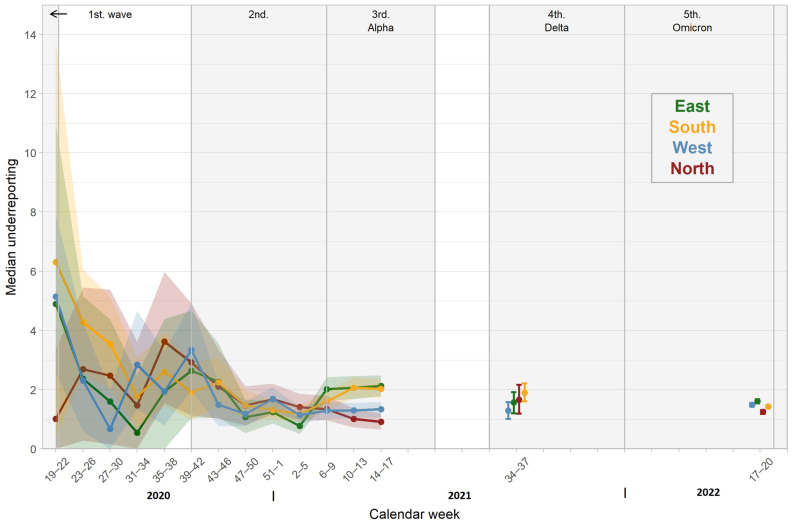
Estimated number of SARS-CoV-2 infections per reported COVID-19 case (underreporting) over time in the different regions.

Stratifying these results for the four study regions, underreporting was significantly higher in the East and the South compared to the North and the West in CW 10–17 in 2021 (Figure 7).

### 3.6. Comparison of Seroprevalence Data with Data from Studies with Different Sampling Approaches

We compared our data with data from other studies in Germany that followed a random sampling approach and had comparable methodology. We used data from the following published studies:

1. SERODUS measured seroprevalence in young adults (aged 18–30 years) in Düsseldorf [25] and we compared it to the results from 18–29-year-old SeBluCo participants in the region “West”. SERODUS used a random sampling method using the population registry and tested samples with the Elecsys Anti-SARS-CoV-2 immunoassay, targeted against nucleocapsid (Roche Diagnostics, Mannheim, Germany). Data were adjusted for test performance and weighted for population characteristics.
2. MusPAD, which measured seroprevalence in the general population in the region of Reutlingen, Freiburg, Aachen, Osnabrück, Magdeburg, and Chemnitz [3]. MusPAD used a random sampling method using the population registries and the S1 IgG ELISA (EUROIMMUN, Lübeck, Germany). Participants were 18–79 years of age and were compared to SeBluCo participants aged 18–65 years. Data were adjusted for test performance and weighted for population characteristics.
3. The nationwide representative RKI-SOEP study (wave 1) used the S1 IgG ELISA (EUROIMMUN, Lübeck, Germany) with dried blood spots [26]. Data were compared for the subset of 18–69-year-old RKI-SOEP participants to 18–65-year-old SeBluCo participants (all regions). Data were adjusted for test performance and weighted for population characteristics.

We compared the published data of these studies with SeBluCo-data from corresponding time periods. In some cases, two SeBluCo time periods are given as one did not match the time frame of the compared study optimally. The data are summarised in Table 3.

## 4. Discussion

Monitoring the SARS-CoV-2 pandemic is essential to effectively implement public health measures. Serosurveys identify infections in the broad range of asymptomatic infected to symptomatic patients and therefore complement surveillance data from notification systems of confirmed cases.

The SeBluCo study monitored SARS-CoV-2 antibodies in residual blood donation specimens collected in 14 of 16 federal states in Germany every two weeks over a period of one year from late April 2020 to April 2021, supplemented by additional samples in September 2021 and April/May 2022. Until early December 2020, the overall estimated seroprevalence in blood donors was less than 2%. This demonstrated the effective containment of the pandemic during the first two waves, which was facilitated by the very strict public health measures but also left the vast majority of 18–65-year-old individuals susceptible to infection prior to vaccination. Starting in mid-December 2020, infection-induced seroprevalence rose moderately and in April 2021, 6.6% of the blood donor specimens showed humoral evidence of infection with SARS-CoV-2. In September 2021, this was the case for 8.6% of donors, and in April/May 2022, for 47.7% of donors. Additionally, free-of-charge vaccination started in late December 2020, and total seroprevalence rose to 18.1% in April 2021, 89.4% in September 2021, and to 100% in April/May 2022. 

Neutralising capacity of the detected antibodies was detected in 74% of positive specimens until April 2021. Since most SARS-CoV-2 infections in blood donors are likely to be mild or even asymptomatic, this proportion is to be expected, especially before the widespread introduction of vaccinations [27]. In the analysed subset of specimens with infection-induced antibodies in April/May 2022, the neutralising capacity was substantially higher reaching 92% for the Omicron variant and 98% for the wild-type virus. Similar proportions of neutralising titres were also observed in studies with donors of convalescent plasma [28].

In our study, we describe the differences in antibody prevalence in geographic regions of Germany. In the first wave of the pandemic, the South was predominantly affected by SARS-CoV-2 infections, and consequently, the detected seroprevalence was higher than in all other regions until the end of July 2020 (CW 31). By the end of the second wave, seroprevalence was similar in all regions. This changed in 2021, when a significantly higher proportion of participants tested positive for infection-induced antibodies in the East and South compared to the West and North. This reflected the higher cumulative incidence in these regions. This was accompanied by a higher degree of underreporting in these regions in CW 9–17 in 2021 as well. The reasons for this remain unclear. Implementation of mitigating measures was performed regionally, but the degree of compliance with these measures on a regional level is unknown. In this context, blood donor surveillance was able to draw a fairly accurate picture of the spread of infection, supplementing the reported incidence. Interestingly, the relatively lower total antibody prevalence in the East in September 2021—which was mainly due to a reduced vaccination rate—may explain in part the early and large spike of infections in these regions in the fourth wave of the pandemic. Repetitive sampling could have been used to target specific mitigation measures such as intensified vaccination campaigns in regions with lower total antibody prevalence. This result underlines the benefit of blood donor samples for serosurveillance, especially if performed repetitively and on a regional level like it has been performed in other countries [10,12,29].

We found that donors aged 18–29 had more infection-induced antibodies than older age groups. This was also the case in most studies with different sampling approaches [3,25] and resembled the age distribution of notified cases in the study period [30]. 

Vaccination was initially offered primarily to the most vulnerable groups [31] and was well accepted by eligible blood donors. Compared to the general population, vaccine coverage in 18–59-year-old blood donors—defined as positive for antibodies against spike protein but negative for antibodies against nucleocapsid—showed no significant difference compared to the general population until April 2021. It was striking, though, that significantly more female donors had vaccine-induced antibodies in March and April 2021 than male donors. This was also observed in the general population [32] and can most likely be attributed to the fact that the prioritised groups for vaccination included caretakers and healthcare workers who are predominantly female [33]. From 24 June 2021 onwards, everyone was eligible to receive a vaccination, and in the cross-sectional sample taken in September 2021, significantly more 18–59-year-old blood donors had measurable antibodies due to vaccination than individuals in the general population, according to the anonymised immunisation registry DIM [22]. Although the DIM probably underestimated the vaccination rates because, e.g., vaccinations by company medical officers were not completely included [34], a difference in vaccination uptake between blood donors and the general population could also be attributed to the so-called “healthy donor effect” [35]. This states that blood donors are generally healthier than the general population and possibly adhere more to health recommendations. Despite the high total seroprevalence in September 2021 (89.4%), a certain proportion of the population was still susceptible to infection, especially with circulating virus variants that could escape immunity. In fact, a modelling study based on weekly incidences of notified SARS-CoV-2 infections, vaccine uptake and assumptions regarding under-ascertainment estimated that 3.8% [quartile range 1.6–5.9%] of 18–59-year-olds in Germany have neither been in contact with vaccine nor any variant up to 31 May 2022 [36].

The degree of underreporting was moderate in the beginning, reaching a factor of five, but quickly dropped to values below two by the end of October 2020, where it remained for the rest of the study period. This underreporting factor was similar to those studies with a representative sampling approach in various regions at the beginning of the pandemic, ranging from 2.2–6.1 [3,6,25]. The reduction in the underreporting factor was most likely due to greater awareness and extensive testing capacities, which were part of the successful strategy to contain the pandemic.

Blood donors can serve as a convenience sample for seroprevalence studies, especially if large numbers of blood specimens from various geographical regions are needed rapidly. By October 2021, they already comprised 22% of all seroprevalence studies performed in a global meta-analysis [15]. Comparing our study with those from countries with comparable mitigation measures and who chose a similar methodological approach, the results are remarkably similar [10,12,37,38]. In contrast, data from Sweden, where mitigation measures were not strictly enforced, show a substantially higher seroprevalence in blood donors, reaching an estimate of 14.8% in December 2020 [39]. This revealed the comparably high seroprevalence in Sweden at the time.

We compared our data to those from studies with a representative sampling approach and a similar methodology and found concordance. Especially the nearly identical weighted and adjusted seroprevalence found in the nationwide RKI-SOEP study [26] supported our view that blood donor samples can indeed contribute in a valuable way to SARS-CoV-2 serosurveillance in the adult population. But also, the comparison of our data with the SERODUS study in young adults [25] showed similar seroprevalences. The MusPAD study [3] was a large, repetitive seroprevalence study in specific regions in various parts of Germany. These results were not readily comparable to our data due to different age groups, size of the regions, and slightly different time periods. Still, we only identified differences in seroprevalence at two sampling points. These results demonstrate that in order to interpret seroprevalence estimates for public health purposes, various sampling approaches should be considered.

Our study has limitations. Blood donors only represent a subset of the healthy adult population. It can be assumed that they were more likely to adhere to non-pharmaceutical interventions during the pandemic than the general population due to the “healthy donor effect” [34]. However, this lack of formal representativeness can be regarded as less important when monitoring the spread of highly contagious pathogens with low population immunity than for non-infectious diseases or infectious diseases with low transmissibility. Still, certain groups of the adult population will most likely be underrepresented in this sample, such as people in care or migrants who are not eligible to donate according to the current Haemotherapy Guidelines [40].

The cross-sectional sampling approach led to a small but non-plausible decrease in the estimated seroprevalence in the summer of 2020 and the beginning of 2021. We can only speculate that this was due to a different group of less exposed blood donors contributing to the sample during these time periods with public and school holidays. Still, the overall trend of the seroprevalence over time was consistent. Naturally, cross-sectional sampling is inferior to sampling in cohorts, but these were not readily available at the beginning of the pandemic.

The specimens were completely anonymised, and therefore, no additional information on infections or vaccinations of participants was available to complement our laboratory-based estimations of infections. But given the fact that individuals might not have been aware of asymptomatic infections or did not seek testing, the estimation can be considered accurate after correcting for test performance. The adjustment for test performance also accounted for a possible waning of antibodies over time because, in our mathematical adjustment, we did not use a single sensitivity and specificity but instead based the correction on a distribution of values for sensitivity and specificity over time, which were available up until 430 days after infection [18]. As N antibodies are more prone to waning, estimates for infection-induced seroprevalence might have underestimated the true prevalence in 2022, more than 2 years into the pandemic.

The strength of our study clearly is the frequent, repetitive sampling in a large number of regions in Germany for more than a year. This gave us the chance to support the constant monitoring of the pandemic and to contribute the data needed for the implementation of public health preventive measures. We were able to demonstrate that, especially in 2020, the mitigation measures in Germany were highly effective and the seroprevalence remained low. We were able to determine the proportion of individuals still susceptible to an infection on a regional level over time and also confirm shortfalls in vaccination uptake in some regions in Germany. These data can also be used to model key indicators such as underreporting and the infection fatality rate. Blood donor specimens are readily available even during lockdowns and can and should be used to support surveillance [12]. This is especially important when emerging infections arise and the need for data is urgent. Blood donors can be an ideal population for infectious disease surveillance under defined circumstances and blood services can partner with public health authorities for informed decision making [41]. Recently, key indicators have been identified to reasonably enable these partnerships [42]. Both public health institutes and blood services should identify ways to cooperate and identify areas in which infectious disease surveillance can be supported by blood donor samples, not only during pandemics.

## Figures and Tables

**Table 1:** Infections waves in Germany in the study period.

Wave	Calendar Week/Year	Virus/Variant
1	10/2020–20/2020	Wild type
2	40/2020–08/2021	Wild type
3	09/2021–23/2021	Alpha
4	31/2021–51/2021	Delta
55	52/2021–21/2022	Omicron

**Table 2:** Total adjusted seroprevalence, infection-induced seroprevalence, and vaccine-induced prevalence stratified for sex over time.

		Total Seroprevalence				Infection-Induced Seroprevalence				Vaccination-Induced Seroprevalence			
Year	CW	Male Donors		Female Donors		Male Donors		Female Donors		Male Donors		Female Donors	
		Prevalence (%)	95% CI	Prevalence (%)	95% CI	Prevalence (%)	95% CI	Prevalence (%)	95% CI	Prevalence (%)	95% CI	Prevalence (%)	95% CI
2020	19–22	1.6	0.7–2.9	0.9	0.1–1.2	1.6	0.7–2.9	0.9	0.1–1.2	No vaccine available			
	23–26	0.8	0.5–1.2	0.8	0.4–1.3	0.8	0.5–1.2	0.8	0.4–1.3				
	27–30	0.6	0.3–1.0	0.6	0.2–1.0	0.6	0.3–1.0	0.6	0.2–1.0				
	31–34	0.5	0.2–0.8	0.8	0.4–1.2	0.5	0.2–0.8	0.8	0.4–1.2				
	35–38	1.2	0.8–1.7	0.5	0.1–0.8	1.2	0.8–1.7	0.5	0.1–0.8				
	39–42	1.2	0.9–1.6	1.1	0.6–1.6	1.2	0.9–1.6	1.1	0.6–1.6				
	43–46	1.7	1.3–2.1	0.8	0.5–1.3	1.7	1.3–2.1	0.8	0.5–1.3				
	47–50	2.0	1.6–2.5	1.5	1.0–2.0	2.0	1.6–2.5	1.5	1.0–2.0				
	51–01	3.2	2.6–3.7	3.7	3.0–4.4	2.9	2.4–3.4	3.4	2.8–4.1	0.3	0.1–0.4	0.3	0.1–0.5
2021	02–05	4.4	3.8–5.1	4.3	3.6–5.1	3.2	2.6–3.8	3.0	2.4–3.7	1.2	0.9–1.6	1.3	0.9–1.7
	06–09	7.5	6.7–8.3	9.5	8.5–10.6	4.8	4.1–5.5	5.1	4.3–5.9	2.7	2.2–3.2	4.4 *	3.7–5.2
	10–13	10.3	9.3–11.2	14.9	13.6–16.2	5.5	4.8–6.3	6.1	5.1–7.0	4.7	4.1–5.4	8.8 *	7.8–9.7
	14–17	14.6	13.5–15.6	21.6	20.1–23.1	6.7	5.9–7.5	7.0	6.0–8.0	8.0	7.3–8.8	14.6 *	13.4–18.8
	36–37	89.1	87.9–90.4	89.6	88.1–91.2	9.0	7.8–10.3	8.2	6.9–9.6	80.1	78.6–81.7	81.4	79.7–83.1
2022	17–20	99.9	99.7–100	100	99.8–100	47.8	46.5–49.1	47.5	46.1–49.1	47.8	46.5–49.1	47.5	46.1–49.1

* Significantly higher than prevalence in male donors.

**Table 3:** Comparison of weighted and adjusted seroprevalence data from different studies in Germany (MusPAD, SERODUS, SOEP) with data from SeBluCo.

Year	CW	Study	Region	Adjusted and Weighted Total Seroprevalence (%; 95% CI)
2020	27–31	MusPAD	Reutlingen	2.0 (1.0–3.0)
	27–30	SeBluCo	South	1.5 (0.8–2.1)
	32–35	MusPAD	Freiburg	1.2 (0.5–1.9)
	31–34	SeBluCo	South	0.8 (0.3–1.3)
	36–40	MusPAD	Aachen	2.0 (1.0–2.9)
	35–38, 39–42	SeBluCo	West	0.8 (0.3–1.2), 1.5 (0.9–2.2)
	41–44	MusPAD	Osnabrück	1.1 (0.4–1.7)
	43–46	SeBluCo	North	0.9 (0.3–1.4)
	41–44	MusPAD	Reutlingen	2.1 (1.2–3.2)
	43–46	SeBluCo	South	1.8 (1.2–2.5)
	45–48	MusPAD	Magdeburg	2.0 (1.1–2.9)
	43–46, 47–50	SeBluCo	East	1.2 (0.5–1.9), 1.3 (0.7–2.0)
	45–48	MusPAD	Freiburg	2.0 (1.0–3.1)
	43–46, 47–50	SeBluCo	South	1.8 (1.2–2.5), 2.3 (1.6–3.1)
	45–48	SERODUS	Düsseldorf (city)	3.1 (2.4–4.0)
	47–50	SeBluCo	West	3.5 (2.1–4.9)
2021	05–08	MusPAD	Aachen	5.2 (3.6–6.9)
	02–05, 06–09	SeBluCo	West	4.4 (3.5–5.3), 8.5 (7.2–9.9)
	09–13	MusPAD	Osnabrück	3.7 (2.1–5.2)
	10–13	SeBluCo	North	11.8 (10.1–13.4) ^†^
	09–13	MusPAD	Chemnitz	14.3 (12.0–16.5)
	10–13	SeBluCo	East	14.7 (12.6–16.8)
	14–17	MusPAD	Magdeburg	9.2 (6.9–11.6)
	14–17	SeBluCo	East	19.1 (16.8–21.5) ^†^
2020/2021	41–08 *	SOEP	nationwide	1.9 (1.3–2.7)
2020	47–50	SeBluCo	nationwide	1.8 (1.4–2.2)

* Median sampling date 11 November 2020. ^†^ CI not overlapping.

## Data Availability

The data set underlying the findings will be available from the Ze-nodo repository.

## Supplementary Materials

The following supporting information can be downloaded at: https://www.mdpi.com/article/10.3390/pathogens12040551/s1, Information S1: Additional statistical information; Table S1: Number of included specimens per time interval.; Table S2: Unadjusted seroprevalence for all age groups (18–83 years).
