# Supplementary material for: Monitoring the SARS-CoV-2 Pandemic: Prevalence of Antibodies in a Large, Repetitive Cross-Sectional Study of Blood Donors in Germany—Results from the SeBluCo Study 2020–2022
Source: Pathogens. 2023 Apr 2;12(4):551. doi: 10.3390/pathogens12040551 (PMC10144823; doi:10.3390/pathogens12040551)
Supplement: Supplementary file 1 [file pathogens-12-00551-s001.zip › pathogens-2283392-supplementary.pdf]

Supplementary information for the article on "Monitoring the SARS-CoV-2 Pandemic: Prevalence of Antibodies in a Large, Repetitive Cross-Sectional Study of Blood Donors in Germany—Results from the SeBluCo Study 2020–2022".

Additional statistical information:

To ensure that each BE contributed consistently over the study period, we estimated the median sample size by age and sex stratum for each BE. Using a bootstrap procedure with 10^3 realisations, we sampled this median sample size from all collected blood specimens with repetition, so that each BE contributed consistently in the respective catchment area to the prevalences of its region by the typically reached sample size. Since the greater regions included six to eight BE this ensured that the minimum sample size was reached in every age and sex stratum.

Since we observed a seroprevalence in the order of one percent in the beginning of the study, we defined a minimum sample size in the following way to assure a good precision of the estimated seroprevalence: Given a true seroprevalence of 1.3%, the binomial probability to observe no positive specimen in the whole random sample should be below 5%. This is true for a sample size of at least 230 specimens. This minimum sample size was hence required for each age and sex stratum by four-week period and region.

To achieve this for a four-week period and a given sex and age group, for which the sample size of a particular BE was slightly below the respective median number (fixed tolerance limit of three samples per four-week period and strata), we drew a bootstrap sample of the median number from this random sample. If the sample size was clearly (more than four specimens) below the median, the missing number of specimens was then randomly drawn from the nearest eligible four-week periods (of the same BE, sex and age group) before and after the considered period. Periods were eligible for topping up reduced sample sizes of their neighbouring period, if they had reached the median number of samples minus the tolerance threshold. Samples drawn from neighbouring eligible periods was realised proportional to the timely distance from the considered time period. In the case of an isolated period with low sample size both directly neighboring periods contributed equally. If the sample size was low in two adjacent time periods, T1 and T2, then for T1 the earlier period T0 contributed with a probability of 2/3 to the topping up, whereas T3 contributed only with a probability of 1/3. For the second period T2 the sampling probabilities were reversed and T0 contributed with a probability of 1/3 and T3 with 2/3. This completion of each random sample size was not performed for the first weeks of the study, since here most BE were only able to test a small number of blood specimens. For April 2021 the random samples with low numbers of specimen were completed using only the first subsequent eligible period in case there was no preceding eligible period. The specimen tested in September 2021 and April/May 2022 were sampled in the quantity they were collected, as no neighboring time period was available. Fortunately, for these periods the sampling of blood specimens was rather complete in all BE and covered the age and sex groups well.

Within this bootstrap we also imputated missing data (<1%) based on logistic regression models built with the original data, but separately for each sampling period and greater geographical region. The estimated

infection and calculated vaccine-induced prevalences were then aggregated to larger units, e. g. whole Germany, using the population numbers of the combined catchment areas in the regions by age group and sex.

Table S1. Number of included specimens per time interval.

Year	Calendar Week	Number of Specimens included
2020	15–18	937
	19–22	5,795
	23–26	9,699
	27–30	10,053
	31–34	10,205
	35–38	9,987
	39–42	9,989
	43–46	6,719
	47–50	7,048
	51–01	6,743
2021	02–05	9,559
	06–09	9,749
	10–13	9,388
	14–17	9,511
	36–37	5,094
2022	17–20	14,034

Table S2. Unadjusted seroprevalence for all age groups (18-83 years.).

Year	Calendar week	Unadjusted total seroprevalence (S1-antibodies)
2020	19-22	1.0
	23-26	1.1
	27-30	0.9
	31-34	0.8
	35-38	1.1
	39-42	1.3
	43-46	1.6
	47-50	2.0
	51-01	3.0
2021	02-05	4.1
	06-09	9.0
	10-13	11.3
	14-17	16.8
	36-37	87.2
2022		
	17-20	98.9
